# Increased chemokine signaling in a model of HIV1-associated peripheral neuropathy

**DOI:** 10.1186/1744-8069-5-48

**Published:** 2009-08-12

**Authors:** Sonia K Bhangoo, Matthew S Ripsch, David J Buchanan, Richard J Miller, Fletcher A White

**Affiliations:** 1 Molecular Pharmacology, Northwestern University, Chicago IL USA; 2 National Institute of Dental and Cranial Research, National Institutes of Health, Bethesda, MD, USA; 3 Cell Biology, Neurobiology & Anatomy, Loyola University, Chicago, Maywood, IL USA; 4 Neuroscience Graduate Program, Loyola University, Chicago, Maywood, IL USA; 5 Anesthesiology, Loyola University, Chicago, Maywood, IL USA

## Abstract

Painful distal sensory polyneuropathy (DSP) is the most common neurological complication of HIV1 infection. Although infection with the virus itself is associated with an incidence of DSP, patients are more likely to become symptomatic following initiation of nucleoside reverse transcriptase inhibitor (NRTI) treatment. The chemokines monocyte chemoattractant protein-1 (MCP1/CCL2) and stromal derived factor-1 (SDF1/CXCL12) and their respective receptors, CCR2 and CXCR4, have been implicated in HIV1 related neuropathic pain mechanisms including NRTI treatment in rodents. Utilizing a rodent model that incorporates the viral coat protein, gp120, and the NRTI, 2'3'-dideoxycytidine (ddC), we examined the degree to which chemokine receptor signaling via CCR2 and CXCR4 potentially influences the resultant chronic hypernociceptive behavior. We observed that following unilateral gp120 sciatic nerve administration, rats developed profound tactile hypernociception in the hindpaw ipsilateral to gp120 treatment. Behavioral changes were also present in the hindpaw contralateral to the injury, albeit delayed and less robust. Using immunohistochemical studies, we demonstrated that MCP1 and CCR2 were upregulated by primary sensory neurons in lumbar ganglia by post-operative day (POD) 14. The functional nature of these observations was confirmed using calcium imaging in acutely dissociated lumbar dorsal root ganglion (DRG) derived from gp120 injured rats at POD 14. Tactile hypernociception in gp120 treated animals was reversed following treatment with a CCR2 receptor antagonist at POD 14. Some groups of animals were subjected to gp120 sciatic nerve injury in combination with an injection of ddC at POD 14. This injury paradigm produced pronounced bilateral tactile hypernociception from POD 14–48. More importantly, functional MCP1/CCR2 and SDF1/CXCR4 signaling was present in sensory neurons. In contrast to gp120 treatment alone, the hypernociceptive behavior associated with the injury plus drug combination was only effectively reversed using the CXCR4 antagonist AMD3100. These studies indicate that the functional upregulation of CCR2 and CXCR4 signaling systems following a combination of gp120 and an NRTI are likely to be of central importance to associated DSP and may serve as potential therapeutic targets for treatment of this syndrome.

## Background

Peripheral neuropathy is the most common neurological complication associated with HIV1 infection. The most common form of neuropathy is a sensory polyneuropathy or HIV1 sensory neuropathy (HIV-SN). HIV-SN can be subdivided into distal sensory polyneuropathy (DSP) and antiretroviral induced toxic neuropathy (ATN). Both forms involve sensory loss and neuropathic pain. DSP occurs in up to 35% of HIV1 infected individuals, while ATN develops following highly active antiretroviral therapy (HAART) treatment in up to 52% of patients [1].

The mechanisms underlying HIV-SN remain unclear. While evidence of neuronal infection by HIV1 is lacking, it is well known that components of the virus such as the coat protein gp120 can bind to, and signal via, neuronal CXCR4 or CCR5 chemokine receptors [2]. Chemokines are well known to direct leukocyte trafficking during inflammatory responses, but numerous studies have now shown additional roles for chemokines that include neural development and modulation of nervous system responses to injury and disease [3,4]. Furthermore, it is known that peripheral sensory neurons can be strongly excited by chemokines and by gp120 [5-7]. As neuronal excitation is a central feature in chronic pain conditions, it is not surprising that a number of chemokine receptors and their ligands have been implicated in multiple rodent models of chronic hypernociception [8].

Previous reports involving gp120 and the nervous system have suggested that gp120 contributes to neurotoxicity and nociceptive behavior in rodents [9-16]. The events that lead to these effects in the nervous system may be dependent on human CD4 (hCD4) binding and conformational changes in gp120 enabling it to bind to chemokine receptors with high affinity [17]. Alternatively, some of the toxic effects of gp120 may be independent of hCD4 binding and be mediated by alternative mechanisms [18,19].

Proposed mechanisms underlying gp120 induced chronic nociception include spinal gliosis. However, peripheral studies in the rodent have shown that perineural gp120 exposure without the addition of hCD4 is accompanied by nerve pathology (distal degeneration of unmyelinated sensory fibers, decreased fiber density and axonal swelling) and an upregulation of proinflammatory cytokine expression [9,10,15]. Despite several investigations into gp120 associated mechanisms underlying chronic mechanical hypernociception, few studies have examined the role of chemokine receptors in gp120 induced mechanical hypernociception in the presence of hCD4.

Painful peripheral neuropathy associated with the use of nucleoside reverse transcriptase inhibitors (NRTIs), a component of HAART, is clinically quite common [20,21], although the mechanisms underlying this phenomenon are yet to be determined. We previously demonstrated that the NRTI, 2'-3'-dideoxycytidine (ddC) not only produced mechanical hypernociceptive behavior but also upregulated CXCR4 mediated chemokine signaling in glia and neurons present in sensory ganglia. Moreover, an antagonist of the CXCR4 receptor, AMD3100, reversed drug induced mechanical hypernociception [22].

Changes in chemokines are not limited to SDF1 as systemic treatment with ddC, nerve treatment with gp120, or a combination of the two treatments also produces pronounced changes in MCP1/CCL2 in sensory neurons of the DRG [15]. Despite emerging evidence that chemokine signaling is central to NRTI induced mechanical hypernociception, little is known regarding the mechanisms underlying the effects of NRTIs in combination with gp120/hCD4, which would represent a closer approximation to the clinical situation.

In order to answer such questions, in the present series of experiments we have tested the hypothesis that perineural gp120/hCD4 treatment alone produces changes in neuronal chemokine signaling and animal behavior. The combination of both gp120/hCD4 and the NRTI further modifies the effects of perineural gp120/hCD4 on animal behavior and chemokine signaling. We have also pharmacologically validated the associated changes in chemokine signaling and associated alterations in mechanical sensitivity with chemokine receptor antagonists.

## Methods

### Animals

Pathogen-free, adult female Sprague-Dawley rats (150–200 g; Harlan Laboratories, Madison, WI) were housed in temperature (23 ± 3°C) and light (12-h light: 12-h dark cycle; lights on at 07:00 h) controlled rooms with standard rodent chow and water available ad libitum. Experiments were performed during the light cycle. All animals were subjected to behavioral assays prior to treatment and randomly assigned to one of experimental or sham treatment groups. These experiments were approved by the Institutional Animal Care and Use Committee of Loyola University, Chicago. All procedures were conducted in accordance with the Guide for Care and Use of Laboratory Animals published by the National Institutes of Health and the ethical guidelines of the International Association for the Study of Pain. Most animal studies include at least 6–8 animals for treatment groups. The data derived from the initial trial for each experiment was analyzed with Power = 95% to determine if sample sizes must be modified for any particular experiment to achieve statistical significance (P < 0.05).

### Injury Model

Animals were anesthetized with 4% isoflurane and maintained on 2% isoflurane (Halocarbon, River Edge, NJ) in O_2_. For the gp120 paradigm, we performed the injury as done previously, with a few modifications [15]. For all gp120 experiments, rgp120 HIV-1 IIIB (Euk, Immunodiagnostics) was dissolved in buffered sterile saline (pH 7.4) to give a final concentration of 800 ng. This solution was combined with 4 μl recombinant human CD4 (hCD4), a glycoprotein coreceptor for gp120 [23], reconstituted in 0.1% BSA/PBS solution (100 μg/ml). The right sciatic nerve of the rat was exposed at the mid-thigh level under sterile conditions. A sterile polyvinyl acetal (PVAc) sponge (Ivalon, San Diego, CA), 2-mm × 2-mm saturated with the gp120 solution, was placed adjacent to the sciatic nerve. The dermal incision site was closed with 5.0 suture thread. Sham control animals were prepared as described above, but buffered sterile saline was used in place of gp120/hCD4 plus saline.

For the gp120/hCD4 in combination with the NRTI model, animals underwent the same procedure described above. At post-operative day (POD) 14, the animals were given a single intraperitoneal (i.p.) injection of the NRTI, 2',3'-dideoxycytidine (ddC, 25 mg/kg; Sigma) freshly prepared in saline. This dose was found to be effective in a previous study (Bhangoo et al., 2007b). Vehicle injections were single i.p. injections of saline.

### Drugs and method of administration

A CCR2 receptor antagonist and its inactive enantiomer, both gifts of Eli Lilly and Co, were employed in this study [24]. The CCR2 antagonist active enantiomer's full name is (R)-4-Acetyl-1-(4-chloro-2-fluorophenyl)-5-cyclohexyl-3-hydroxy-1,5-dihydro-2H-pyrrol-2-one (CCR2 RA **[R]**), and it was used as a Na+ salt. Some animals were given a saline vehicle injection. Both compounds were freshly prepared in saline on the day of the experiment (10 mg/kg). Receptor antagonist and vehicle-treated groups (n = 8 per group) were given a single i.p. injection one hour prior to behavioral testing. The bicyclam, AMD3100 (5 mg/kg, Sigma), was also used. These treated animals were also given a single i.p. injection of this CXCR4 receptor antagonist one hour prior to behavioral testing. Antagonist dosages were determined in previous studies [22,24].

### Foot withdrawal to punctate mechanical indentation

The incidence of foot withdrawal was measured in response to mechanical indentation of the plantar surface of each hindpaw with Von Frey-type filaments. Mechanical stimuli were applied with seven filaments, each differing in the bending force delivered (10, 20, 40, 60, 80, 100, and 120 mN). Each filament was fitted with a flat tip and a fixed diameter of 0.2 mm [22,25-27]. The force equivalence of mN to grams is: 100 mN equals 10.197 g.

The rat was placed on a metal mesh floor and covered with a transparent plastic dome. Typically, the animals rest quietly in this situation after an initial few minutes of exploration. Animals were habituated to this testing apparatus for 15 minutes a day, two days prior to the behavioral assays. Following acclimation, each filament was applied to six locations spaced across the hind paw that correspond to the nerve distribution in the glabrous skin. The filaments were tested in order of ascending force, with each filament delivered in sequence from the 1^st ^to the 6^th^location alternating from one hindpaw to the other. The duration of each stimulus was 1 second and the interstimulus interval was 10–15 seconds. A cutoff value of 120 mN was used; animals that did not respond at 120 mN were assigned that value [22,27]. In each behavioral testing sequence, the operator was blinded to the animal treatment condition.

The incidence of paw withdrawal was expressed as a percentage of the six applications of each filament as a function of force. A Hill equation was fitted to the function (Origin version 6.0, Microcal Software, Northhampton MA) relating the percentage of indentations eliciting a withdrawal to the force of indentation. From this equation, the paw withdrawal threshold (PWT) force was obtained and defined as the force corresponding to a 50% withdrawal. At least a -20 mN difference from the baseline PWT in a given animal is representative of mechanical hypernociception [22,27].

Measurements were taken on three successive days before surgery. Postoperative testing was performed on post-operative day (POD) 5, 7, 10, 14 and weekly thereafter for the duration of the experiment. PWT values were statistically analyzed for each foot separately and for the significance of differences between the average of the three preoperative tests and the mean obtained for each postoperative test. The same statistical analyses are applied to the slopes of the logistic functions from which the PWTs were derived. The experimenter was blinded to both the injury condition of the animal and the drugs utilized in all behavioral trials.

### Foot withdrawal to thermal stimulus

To evaluate the PWT to thermal stimulation, the Hargreaves' plantar test apparatus (Ugo Basile, Varese, Italy) was used. Rats were placed on a 2-mm-thick glass floor; a mobile infrared heat generator with an aperture of 10 mm was aimed at the rat's hindpaw under the floor. Following activation of the heat source, the reaction time (the withdrawal latency of the hindpaw) of the rat was recorded automatically. A shortening of the withdrawal latency indicated thermal hyperalgesia. The temperature of the glass floor was kept at 22.5–23.5°C. Measurements of the withdrawal latency of the paw began after the rats were habituated to the testing environment (IR setting = 70). The measurements were repeated three times, at 5 min intervals, on each paw. The averages of the three pairs of measurements taken were employed as data.

### In situ hybridization

In situ hybridization histochemistry for chemokine receptors was performed by using digoxigenin-labeled riboprobes. Adult female Sprague-Dawley rats were euthanized using carbon dioxide. L_4_L_5 _DRGs ipsi- and contralateral to LPC nerve injury were rapidly removed, embedded in OCT compound (Tissue Tek, Ted Pella, Inc., Redding, CA) and frozen. Sections were serially cut at 14 μm. The CCR2 probe was prepared as previously described [7]. Briefly, an 848-bp CCR2 cDNA fragment (nucleotides 489–1336 of GenBank no. U77349) was cloned by PCR using rat spleen cDNA. The resulting PCR product was subcloned into a pGEM-T Easy vector and sequenced to ensure identity for riboprobe use. The CCR2 template was linearized with SacII to generate a probe of 950 bases by using SP6 polymerase. Signals were visualized by using NBT/BCIP reagents (Roche Diagnostics/Boehringer Mannheim, Indianapolis, IN) in the dark for 2–20 h depending upon the abundance of the RNA. The CXCR4 and SDF1 probes were generated as described previously [3]. Images were captured using brightfield or differential interference contrast optics with a Nikon E600 fluorescent microscope (NikonUSA, Melville, NY) fitted with a charge-coupled device camera (Retiga EXi, Q-Imaging Corporation, Vancouver, BC).

### Immunohistochemical labeling

Adult female Sprague-Dawley rats were euthanized with CO_2 _and transcardially perfused with saline followed by 4% paraformaldehyde. Lumbar ganglia associated with the sciatic nerve ipsilateral to the nerve injury (n = 6) or sham treatment (n = 6) were immediately removed and post fixed for 4 hours. Additional lumbar DRGs were removed from naïve, behaviorally tested rats (n = 6). Lumbar DRGs were encoded at the outset and processed in random order. Sagittal sections of the DRG were serially cut at 14 μm onto SuperFrost Plus microscope slides (Fisher Scientific, Pittsburgh PA). At least 6 sections were obtained for immunohistochemical analysis per DRG. Tissue was processed such that DRG sections on each slide were at intervals of 80 um. Slides were incubated with blocking buffer (3% serum/0.4% Triton-X; Fisher Scientific, Pittsburgh PA) for 1 hour, followed by overnight incubation with anti-MCP1 (rabbit polyclonal, 1:500; Chemicon, Temecula, CA), CXCR4 (rat monoclonal, 1:20,000; BD Pharmingen) or CCR2 (rabbit polyclonal, 1:500; Aviva Systems Biology, San Diego CA) at room temperature. After primary incubation, slides were incubated in secondary antibodies (goat conjugated to CY2, Jackson ImmunoResearch, West Grove, PA) which were used to visualize cells. For some double-labeling experiments, slides were then incubated in anti-TRPV1 antibody (rabbit polyclonal, 1:1000; Neuromics), followed by incubation in secondary antibodies (donkey conjugated to CY5). Some experiments were augmented with the addition of IB_4 _conjugated with fluorescein (1 mg/1 ml; Sigma, St. Louis MO). Slides were washed in PBS for 5 min each (x3) and coverslipped with a PBS/glycerol solution. All tissue sections were also stained with DAPI nuclear marker (Invitrogen Corporation, Carlsbad CA).

Tissue sections were analyzed for the presence of TRPV1 or IB_4_-binding neurons and either CXCR4 or CCR2. Because a stereological approach was not employed in this study, quantification of the data may represent a biased estimate of the actual numbers of immunopositive neurons. The proportions of immunoreactive neurons were determined from the total number of DAPI-positive neuronal nuclei present in a tissue section. The overall diameter and brightness of the DAPI-positive neuronal nuclei allowed for a clear delineation between neurons and non-neuronal cells in the DRG. A positive cell was considered to be one with clear cytoplasmic staining, and the outline of the nucleus was apparent. At least 2000 neuronal profiles from six animals were quantified for each cell type in the single neuronal marker study and for each combination of cellular markers. Quantification of cell numbers and degree of colocalization was determined using Image J (National Institute of Health). Data are represented as means ± SEM%.

### Preparation of acutely dissociated dorsal root ganglion cells

The L_4_-L_5 _DRG were acutely dissociated using methods described by Ma and LaMotte [28]. Briefly, L_4 _and L_5 _DRG were removed from sham control or treated animals at various post-operative time points. The DRGs were treated with collagenase A and collagenase D in HBSS for 20 minutes (1 mg/ml; Roche Applied Science, Indianapolis, IN), followed by treatment with papain (30 units/ml, Worthington Biochemical, Lakewood, NJ) in HBSS containing .5 mM EDTA and cysteine at 35°C. The cells were then dissociated via mechanical trituration in culture media containing 1 mg/ml bovine serum albumin and trypsin inhibitor (1 mg/ml, Sigma, St. Louis MO). The culture media was equal amounts of Ham's F12 mixture and DMEM supplemented with 10% fetal bovine serum and penicillin and streptomycin (100 ug/ml and 100 U/ml). The cells were then plated on cover slips coated with poly-L-lysine and laminin (1 mg/ml) and incubated for 2 hours before more culture media was added to the wells. The cells were then allowed to sit undisturbed for 12–15 hours to adhere at 37°C (with 5% CO_2_).

### Intracellular Ca^2+ ^imaging

The dissociated DRG cells were loaded with fura-2 AM (3 uM, Molecular Probes/Invitrogen Corporation, Carlsbad CA) for 25 minutes at room temperature in a balanced salt solution (BSS) [NaCl (140 mM), Hepes (10 mM), CaCl_2 _(2 mM), MgCl_2 _(1 mM), Glucose (10 mM), KCl (50 mM)]. The cells were rinsed with the BSS and mounted onto a chamber that was placed onto the inverted microscope and continuously perfused with BSS at a rate of 1 ml/min. Intracellular calcium was measured by digital video microfluorometry with an intensified CCD camera coupled to a microscope and MetaFluor software (Molecular Devices Corporation, Downington, PA). Cells were illuminated with a 150 W xenon arc lamp, and the excitation wavelengths of the fura-2 (340/380 nm) were selected by a filter changer. Chemokines were applied directly into the cover slip bathing solution after the perfusion was stopped. If no response was seen within 1 minute, the chemokine was washed out. For all experiments, interferon-γ-induced protein 10 (IP-10/CXCL10), MCP1 and SDF1 were added to the cells in random order, after which capsaicin, high K+ (50 K) and ATP were added. The chemokines used were purchased from R & D Systems (Minneapolis, MN), and all were used at a concentration of 100 nm to ensure maximal activation. They were reconstituted in 0.1%BSA/PBS, and aliquots were stored at -20°C. A minimum of 50 neurons was analyzed for each chemokine.

### Statistical Analyses

Data is presented as the mean ± SEM, unless otherwise noted. GB-Stat School Pack software (Dynamic Microsystems, Inc. Silver Springs, MD) and Graphpad Prism (GraphPad Software, Inc.) were used to statistically evaluate all data. Significant differences were determined by one-way ANOVA with Bonferroni's post-hoc test for animal behavior. A one-way ANOVA with a Dunnett's Multiple Comparison test was used to analyze the differences between naïve, sham and experimental groups in the fura imaging studies. A difference of p < 0.05 was considered significant.

## Results

### Perineural gp120/hCD4 treatment produces mechanical hypernociception in the hindpaw ipsilateral to the injury

**Figure 1:**
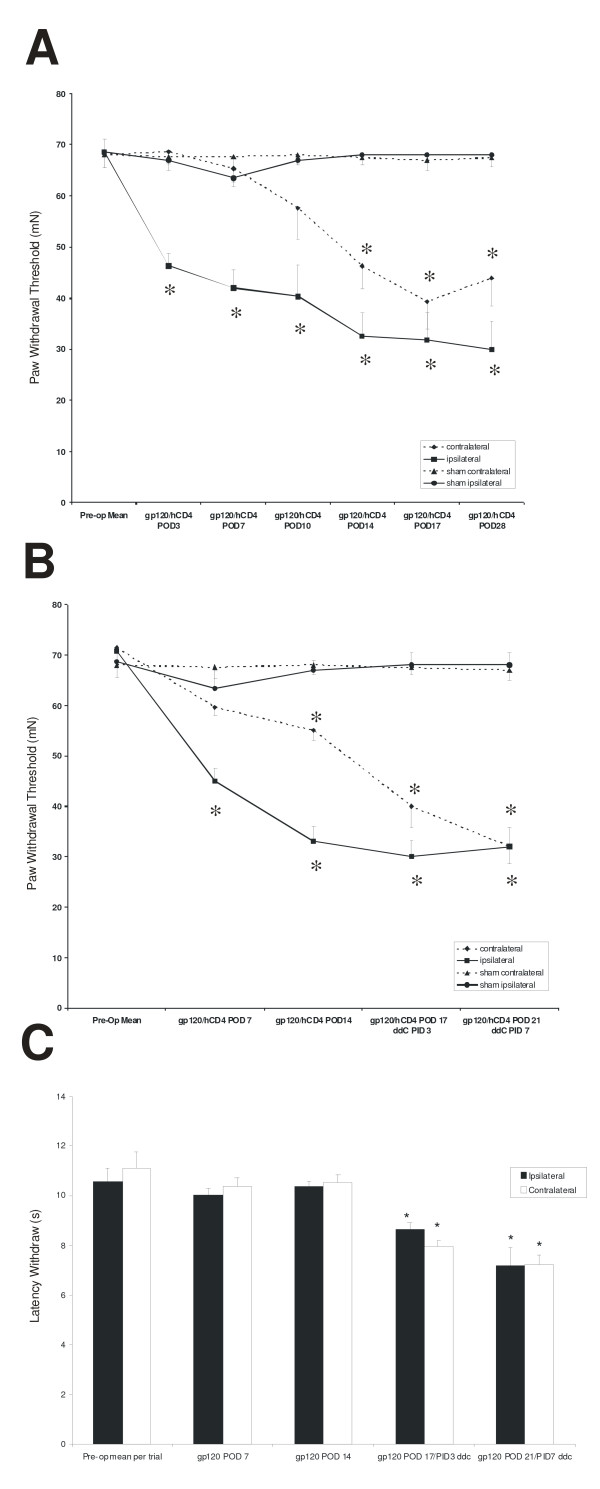
**Mechanical and thermal behavioral responses following perineural gp120/hCD4 treatment alone or in combination with a single 25 mg/kg i.p injection of 2'-3'-dideoxycytidine**. **A**) Rats were assessed for low-threshold mechanical sensitivity (von Frey test) at 3, 7, 10, 14, 17, 28 days following perineural gp120/hCD4 treatment. Reduced behavioral thresholds for the hindpaw ipsilateral to the nerve lesion (black square) were significantly different from pre-operative baseline from post-operative day (POD) 3–28. The threshold force for the hindpaw contralateral to the nerve lesion (black diamond), did not meet the pre-determined criteria indicative of mechanical hypernociceptive behavior until POD17. The time course of sham injury did not differ from the uninjured animals (ipsilateral sham, black circle; contralateral sham, black triangle). (n = 10; * p < 0.01). POD: post-operative day after gp120/hCD4 treatment. **B**) Mean threshold force required for paw withdrawal to Von Frey stimulation following perineural gp120/hCD4 treatment and a single 25 mg/kg i.p injection of 2'-3'-dideoxycytidine (ddC; n = 10). The ddC injection was given on POD14 after gp120 treatment. Reduced behavioral thresholds for the hindpaw ipsilateral (black square) and contralateral (black diamond) to the nerve treatment were seen as early as POD17/post-injection day (PID) 3 and reached the lowest levels by POD21/PID7 (p < 0.01). The time course of sham injury did not differ from the uninjured animals (ipsilateral sham, black circle; contralateral sham, black triangle). **C**) gp120/hCD4 induced nerve injury did not produce changes in thermal responses as assessed by the Hargreaves test. Each bar is the mean withdrawal latency (± SE) of the hindpaw ipsilateral (white bar) or contralateral (black bar) to the nerve injury at POD 7 and 14 (n = 10). After a single i.p. injection of ddC at POD 14, thermal hyperalgesia was observed as early as PID 3 and reached the shortest latency by PID7 in the paws ipsilateral and contralateral to the gp120/hCD4 treatment. POD: post-operative day after gp120/hCD4 treatment; PID: post-injection day after ddc treatment. (n = 10; *p < .05).

After establishing a baseline behavioral assessment, rats were treated with a perineural application of either vehicle (saline), or gp120/hCD4 in saline (*n *= 6–8/group). Rats treated with gp120/hCD4 displayed mechanical hypernociception (-20 mN change in PWT) as measured by graded von Frey filaments in the limb ipsilateral to the injury by POD 5 (Fig. 1A; n = 10, p < 0.001) and in the limb contralateral to the injury by POD 17 (Fig. 1A; n = 10, p < 0.01). The force required to elicit a PWT remained significantly below baseline thresholds until POD 28 for the hindpaw contralateral to the lesion and POD 35 for the hindpaw ipsilateral to the injury. Vehicle treated sham operated rodents did not exhibit a PWT decrease that was significant at any time point (Fig. 1A). Upon completion of testing, rats were euthanized and tissues utilized for further analysis.

### Perineural gp120/hCD4 treatment in combination with ddC produces bilateral mechanical hypernociception

A single i.p. injection of ddC was administered at day 14 after an initial perineural treatment with gp120/hCD4 treatment (n = 8). As opposed to the largely unilateral changes in PWT observed at 17 days following gp120/hCD4 treatment, the addition of the ddC treatment further diminished PWT in both hindpaws. This change in PWT occurred within 3 days of the ddC injection (Fig. 1B; n = 10, p < 0.01). Similar to previous observations [22], the lowest PWTs were exhibited by 7 days post ddC injection. These bilateral injury induced changes lasted for at least 49 days (data not shown).

### Perineural gp120/hCD4 and ddC produce differential effects on thermal thresholds

The thermal PWTs of the gp120/hCD4 treated rats from POD 0–14 (10.38 +/- .17 seconds) did not differ from the baseline threshold (10.57 +/- .55 seconds; Fig. 1C; n = 10, or from vehicle treated rodents (data not shown). In contrast, the thermal withdrawal thresholds of rats treated with a combination of gp120/hCD4 and ddC at POD 14 were reduced to 7.18 +/- .73 seconds when compared with baseline thresholds (10.38 +/- .17 seconds). This difference in the latency withdrawal (-3.2 ± 0.09 s) was significantly different (Fig. 1C; n = 10, *p *< 0.05).

### Injury induced alteration of CCR2 mRNA and protein expression in lumbar DRG

**Figure 2:**
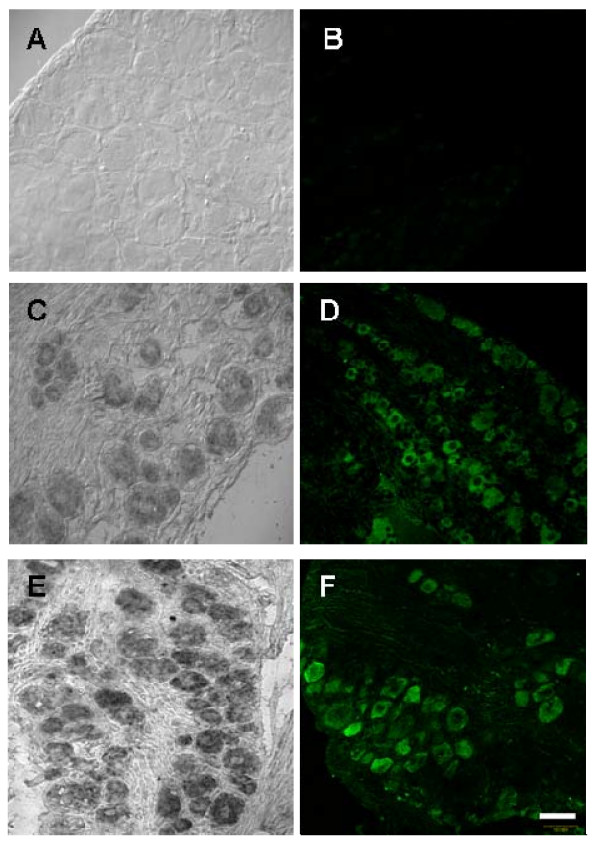
**Expression of CCR2 mRNA and protein immunoreactivity in rat lumbar DRG ipsilateral to gp120/hCD4 nerve treatment and ddC treatment at post-operative day (POD) 14 and 21**. **A**) Lumbar DRG removed from vehicle-treated animals at POD14 did not exhibit CCR2 mRNA expression (n = 3). **B**) There was no evidence of CCR2 protein expression in sham animals (n = 3). **C**) Lumbar DRG neurons from gp120/hCD4 injured rats on POD14 exhibited CCR2 mRNA transcripts in neurons of all sizes (n = 10). **D**) Lumbar DRG neurons from a rat subjected to gp120/hCD4 treatment only exhibited CCR2 immunopositive small and medium diameter sensory neurons (n = 10). **E**) In rats treated with a combination of gp120/hCD4 and ddC and sacrificed at POD21, many lumbar DRG neurons exhibited CCR2 mRNA transcripts. **F**) CCR2 immunoreactivity was also present in numerous neurons at POD21 after combined gp120/hCD4 and ddC treatment. Scale bar: 50 um.

Lumbar DRGs removed from naive failed to exhibit CCR2 immunopositive cells (see additional file 1A) or CCR2 mRNA cells (see additional file 1C). Likewise, vehicle-treated rats at POD 14 failed to exhibit CCR2 mRNA (Fig. 2A) or CCR2 immunopositive cells (Fig. 2B). Following perineural gp120/hCD4, sensory neurons in the lumbar DRG ipsilateral to the focal nerve injury were observed to express CCR2 mRNA transcripts (Fig. 2C) and were immunopositive for CCR2 (Fig. 2D). The neuronal expression of CCR2 mRNA in rodents subjected to both gp120/hCD4 and the NRTI did not differ from gp120/hCD4 treatment alone (Fig. 2E–F). Moreover, the number of CCR2 immunopositive neurons present in gp120/hCD4 treated DRG (27.8 ± 1.9% of total neurons) or in combination with ddC (24.15 ± 1.5% of total neurons) did not exhibit significant differences.

**Figure 3:**
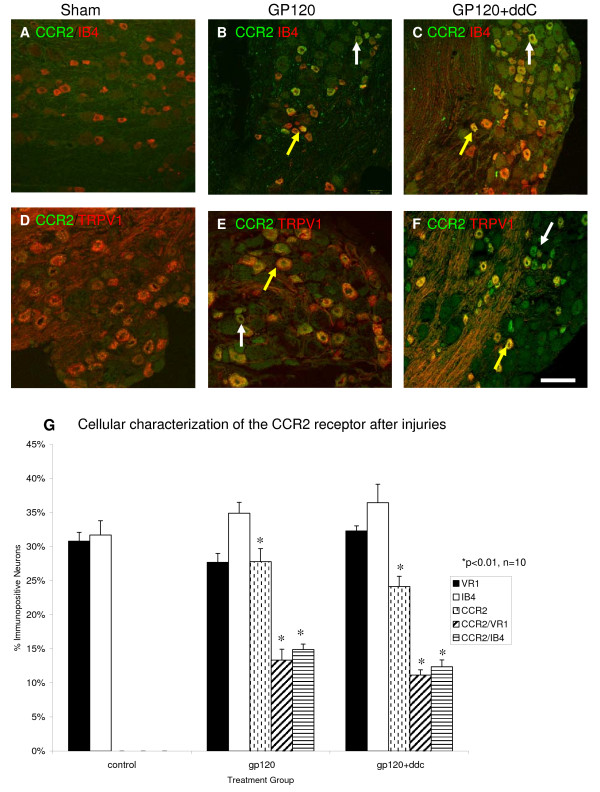
**The CCR2 chemokine receptor colocalized with IB_4 _and TRPV1, markers of nociceptive neurons, after injury**. **A) **Many lumbar DRG neurons in vehicle-treated rat sensory neurons were positive for IB_4_, a neuronal phenotype that distinguishes some C-fiber nociceptors (red cells), however there was no expression of the CCR2 protein. **B) **After perineural gp120/hCD4 treatment, CCR2 protein expression (green cells) was upregulated, and co-localized with IB_4_. **C) **Both gp120/hCD4 and ddC treatment resulted in an upregulation of CCR2 expression (green cells) in many small and medium diameter neurons. Again, CCR2 co-localized in a number of IB_4 _positive cells. **D) **The TRPV1 channel is present on many nociceptive neurons and is involved in the neuropathic pain mechanism. Under normal conditions, TRPV1 was expressed in neurons (red cells). **E) **After gp120/hCD4 treatment, CCR2 expression is upregulated (green cells) and colocalized with TRPV1. **F) **After the combination of gp120/hCD4 and ddC treatments, again CCR2 was upregulated to a similar degree as gp120/hCD4 treatment alone and exhibited some colocalizations with TRPV1. **G) **The percentage of CCR2-positive cells that co-localized with IB_4 _and TRPV1. CCR2 receptor expression was upregulated after gp120/hCD4 alone and the combination of gp120/hCD4 and ddC treatments. Most cells were of small and medium diameter. Approximately half of the CCR2-positive cells also colocalized with IB_4 _or TRPV1. Sham treatment did not result in an upregulation of CCR2 protein expression. IB4 and TRPV1 were expressed in all conditions, and did not differ significantly between groups. Yellow arrows: colocalizing neurons; white arrows: non-colocalizing neurons. Data represents mean ± SE. Scale Bar: 100 um.

In an attempt to further establish the identity of CCR2 immunopositive sensory neurons, we performed double labeling immunofluorescence with well established neurochemical markers of key sensory neuron subpopulations. Neuronal binding of the plant isolectin, IB_4 _in the rat DRG distinguishes a non peptidergic subpopulation of C-fiber nociceptors that project largely to spinal cord dorsal horn lamina II [29,30]. Many IB_4 _binding neurons were present in lumbar DRG of sham operated rats and those subjected to gp120/hCD4 and the combination of gp120/hCD4 and ddC (Fig. 3A, B, C, and 3G). 32 ± 2% of the total neurons were positive for IB_4 _in DRG derived from sham treated rodents. Following gp120/hCD4 treatment, 27.8 ± 1.9% of neurons were immunopositive for CCR2 and approximately half of those cells were colocalized with IB_4 _(53.6 ± 0.8%; Fig. 3G). Sensory neurons in DRG derived from gp120/hCD4 and ddC treated rodents (n = 6) displayed similar numbers of neurons that bound IB_4 _and were CCR2 immunopositive (51.3 ± 1%).

### Coexpression of the TRPV1 receptor channel with CCR2 receptors

We performed double labeling immunofluorescence for the vanilloid receptor TRPV1, a ligand gated cation channel that is responsive to several diverse stimuli and important in nociceptive pain responses. We found that in sham treated tissues, 30.8 ± 1.3% of neurons expressed the TRPV1 protein, while there was no CCR2 expression (Fig. 3D, G). Fourteen days after perineural gp120/hCD4 treatment, the number of neurons expressing TRPV1 had not changed (27 ± 1.7%). However, as mentioned above, numerous CCR2 immunopositive cells were present and nearly half of these cells colocalized with TRPV1 (48 ± 1.6% Fig. 3E, G). The combination of gp120/hCD4 and ddC treatment did not produce significant changes in the percentage of colocalized CCR2/TRPV1 immunopositive neurons when compared with rodents subjected to perineural gp120/hCD4 (46.2 ± 0.78%; Fig. 3F, G).

### Injury induced alteration of CXCR4 mRNA and protein expression in lumbar DRG

In addition to CCR2, other chemokine receptors may also be involved in neuropathic pain mechanisms. To establish the degree to which CXCR4 signaling is evident in rodents subjected to perineural gp120/hCD4 alone or in combination with ddC, we first determined to what extent this chemokine receptor was altered by the same injury paradigms as discussed above. One example of this is SDF1 signaling via CXCR4 receptors. Peripheral nerves constitutively express the chemokine SDF1 and its receptor CXCR4, and basal expression of SDF1 and CXCR4 mRNA in the rat is predominantly detected in non-neuronal cells of the lumbar DRG derived from naïve animals [22]. Subsequent to peripheral nerve injury or ddC treatment both SDF1 and CXCR4 exhibit increased expression [31-33].

**Figure 4:**
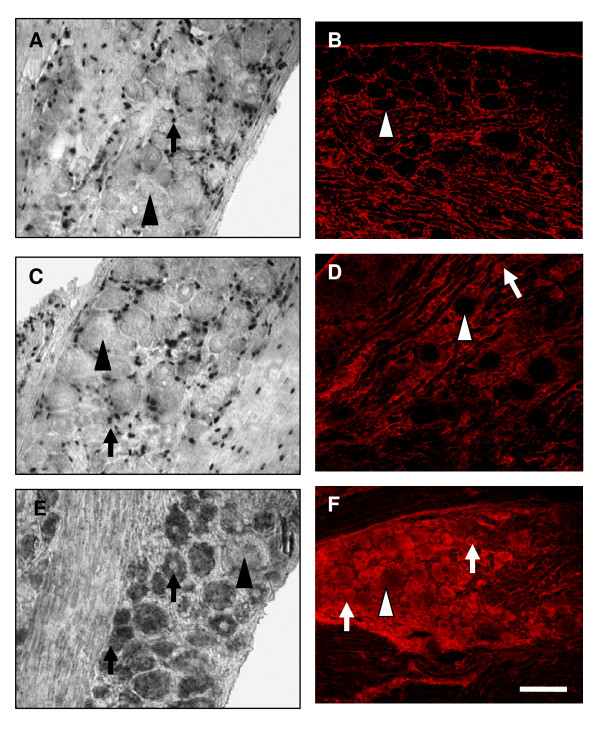
**Expression of CXCR4 in rat lumbar DRG ipsilateral to gp120/hCD4 nerve treatment and following the combination of gp120/hCD4 and ddC treatments**. **A**) CXCR4 mRNA transcripts present in lumbar DRG removed from vehicle-treated rodents at post-operative day (POD)14. Many non-neuronal cells strongly expressed CXCR4 mRNA. This data is not unlike previous observations [22]. **B**) CXCR4 protein levels after sham treatment were mainly expressed in non-neuronal cells and only occasional neurons. After gp120/hCD4 treatment, the mRNA and protein patterns of expression for the CXCR4 receptor did not change by POD14, when compared to sham treated animals **(C,D)**. **E**) gp120/hCD4 treatment, in combination with ddC treatment produced upregulation of CXCR4 mRNA expression in neurons of all sizes by POD21. **F) **Protein expression for the CXCR4 receptor was also upregulated in neurons of mostly small and medium diameters after the combination of gp120/hCD4 and ddC treatments at POD21. Arrows: positive cells; arrowheads: negative cells. Scale bar: 100 um. (n = 4/treatment condition).

Consistent with previous observations, immunohistochemical techniques used in these experiments revealed non-neuronal CXCR4 immunoreactivity present in both naïve (see additional file 1B and 1D) and sham treated sensory neurons (Fig. 4A, B). This level of non-neuronal expression did not change appreciably following vehicle treatment (Fig. 4A, B) or perineural gp120/hCD4 treatment (Fig. 4C, D). In contrast, combined treatment of animals with gp120/hCD4 and ddC produced an increase in the number of neurons expressing CXCR4 protein by POD21 in the lumbar DRG ipsilateral to the nerve lesion. Notably satellite cells under these conditions lacked both CXCR4 mRNA transcripts and protein expression (Fig. 4E, F).

**Figure 5:**
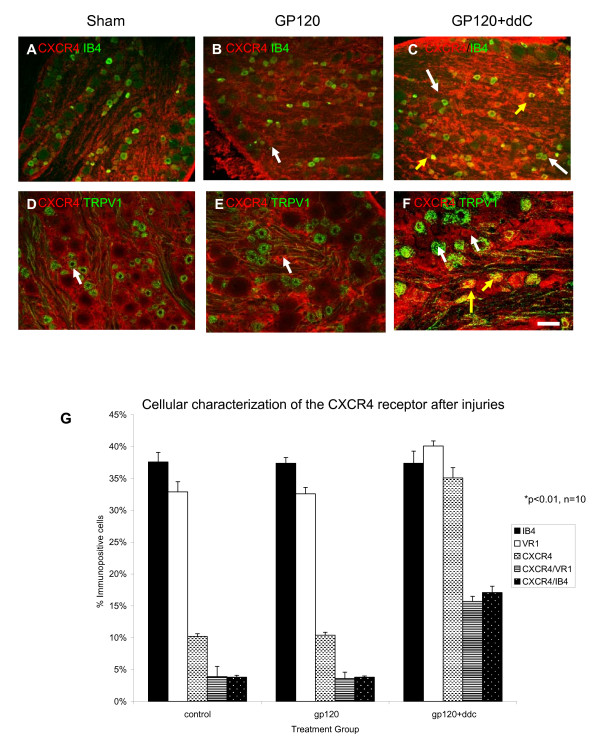
**The CXCR4 chemokine receptor colocalizes with IB_4_-binding and TRPV1-immunopositive neurons following injury**. **A) **Under sham conditions, CXCR4 was expressed in mainly non-neuronal cells, along with occasional neurons. Many lumbar DRG neurons in vehicle-treated rat sensory neurons were positive for IB_4_, a neuronal phenotype that distinguishes some C-fiber nociceptors (green cells), however there was little colocalization of CXCR4 with IB_4_-binding cells. **B) **Fourteen days after gp120/hCD4 treatment, CXCR4 and IB_4 _protein expression (red cells) patterns did not change, when compared to sham treated animals **C) **Both gp120/hCD4 and ddC treatment resulted in an upregulation of CXCR4 expression in many small and medium diameter neurons by POD21. CXCR4 co-localized in a number of IB4-positive cells. **D) **Under normal conditions, TRPV1 was expressed in neurons (green cells). CXCR4 was expressed in non-neuronal cells, and there was little co-localization with TRPV1. **E) **gp120/hCD4 treatment did not produce changes in the distribution or colocalization of CXCR4 and TRPV1. **F) **Numerous neurons exposed to the combination of gp120/hCD4 and ddC treatment exhibited both CXCR4 upregulation and colocalization with TRPV1 by POD21. **G) **The percentage of CXCR4-positive cells that co-localized with IB_4 _and TRPV1. CXCR4 receptor expression was present under sham conditions and did not change following gp120 treatment. Following the combination of gp120/hCD4 and ddC treatment, CXCR4 immunoreactivity was observed approximately half of the IB_4_-binding and TRPV1-immunopositive cells of small and medium diameter. Data represents mean ± SE; p < 0.01, n = 10. Yellow arrows: neuronal colocalization; white arrows: single-labeled neurons. Scale Bar: 50 um.

To further characterize changes in CXCR4 receptor following gp120/ddC treatment, colocalization experiments were performed with IB_4 _and TRPV1. Under normal conditions, there was a basal level of neuronal CXCR4 expression (10.2 ± 0.44%). Colocalization of CXCR4 and either IB_4 _or TRPV1 was limited to only 3.8 ± 0.3% or 3.9 ± 0.13% of the total neurons counted respectively (Fig. 5A, D, G). This is not surprising because the CXCR4 expression seen under these conditions is predominantly observed in non-neuronal cells. gp120/hCD4 treatment did not change the number of colocalized cells (Fig. 5B, E, and 5G).

After the combination of gp120/hCD4 and ddC, there was an increase in the number of CXCR4 immunopositive neurons (35.1 ± 1.6%) (Fig. 5C, F, G). The percentage of total neurons colocalized with either IB_4 _or TRPV1 increased to 17.1 ± 1% and 15.7 ± 8% of total neurons counted, respectively.

### Changes in chemokine expression following perineural gp120/hCD4 alone and in combination with ddC treatment

**Figure 6:**
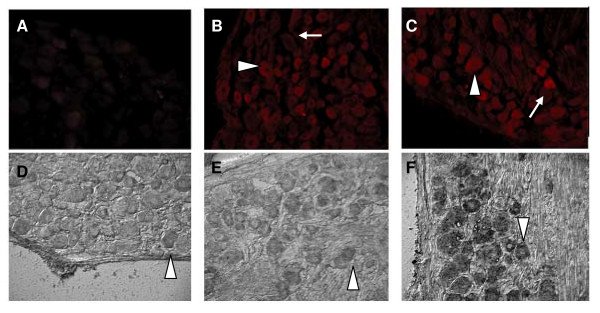
**Chemokine expression after gp120/hCD4 alone and following the combination of gp120/hCD4 and ddC treatment**. **A) **MCP1 expression, the ligand for the CCR2 receptor was absent from sham treated animals. **B) **After gp120/hCD4 treatment, MCP1 expression was upregulated in mostly neurons of the DRG. **C) **After the combined treatments of gp120/hCD4 and ddC, MCP1 expression was upregulated in a similar manner to that seen in the gp120/hCD4 only treatment. **D) **SDF1, the ligand for CXCR4, had a basal level of mRNA expression, with the occasional neuron staining positive for the chemokine **E) **After gp120/hCD4 treatment, the level of SDF1 mRNA expression did not change appreciably, when compared to sham treated animals. **F) **Following gp120/hCD4 and ddC treatments, SDF1 mRNA expression was upregulated in neurons of all sizes. Arrows: positive cells; arrowheads: negative cells. Scale bar: 100 um.

The number of cells exhibiting MCP1 (immunoreactivity) or SDF1 (mRNA transcripts), the respective ligands for the CCR2 and CXCR4 receptors, were also analyzed using the both injury paradigms. Under sham conditions, there were virtually no MCP1 immunopositive neurons in the lumbar DRG (Fig. 6A). In sharp contrast, numerous MCP1 immunopositive neurons were present in lumbar ganglia of the gp120/hCD4 treated rats by POD 14 (Fig. 6B). There was also a complete lack of MCP1 immunopositive non-neuronal cells in injured DRG at any time point.

MCP1 immunopositive neurons were also present in the DRG of animals treated with both gp120/hCD4 and ddC combined (Fig. 6C) as previously observed [15]. The number of MCP1 immunopositive neurons present in the lumbar DRG following the combined injury was similar to numbers present in gp120/hCD4 treatment condition (data not shown).

Previous studies of neuropathic injury in rodents have demonstrated ddC treatment induced changes in SDF1/CXCR4 signaling by neurons [22]. Lumbar DRG in the naïve (data not shown), vehicle treated and gp120/hCD4 only treated rats were largely devoid of the SDF1 mRNA transcripts with the exception of the occasional neuron (Fig. 6D, E). In sharp contrast, following the combination of gp120/hCD4 and ddC injury paradigm, mRNA transcripts were upregulated by POD 21 in the DRG ipsilateral to injury in virtually all neurons (Fig. 6E).

### Chemokines increase [Ca^2+^]i in DRG cells subjected to gp120/hCD4 treatment alone and in combination with ddC

Activation of chemokine receptors expressed by primary sensory neurons results in excitation and in the increase in the intracellular Ca^2+^concentration [5]. Fura-2 based Ca imaging of chemokine induced increases in [Ca^2+^]i in acutely isolated rat DRG neurons was used as a measure of functional chemokine receptor expression. DRG cells were acutely isolated from the ipsilateral side of nerve injured animals and sham controls. For all experiments, the chemokines MCP1, SDF1 and IP-10 were added in random order to the cells, after which capsaicin, high K+(50 mM) and ATP were added to assess the cells' identity and viability, respectively. A response to high K+ stimulation indicates the presence of voltage dependent Ca2+ channels, which is indicative of neurons. Additionally, a positive response to capsaicin as well as high K+ indicates the cell is a nociceptor expressing the TRPV1 channel. If the cell did not respond to either capsaicin or K+, ATP was added to the bathing solution. A response to ATP, which activates purinergic receptors, without one to High K+ and/or capsaicin, indicates a non-neuronal cell, such as a glial cell. The concentrations of chemokines used in these experiments were all supramaximal to ensure activation of any expressed receptors.

**Figure 7:**
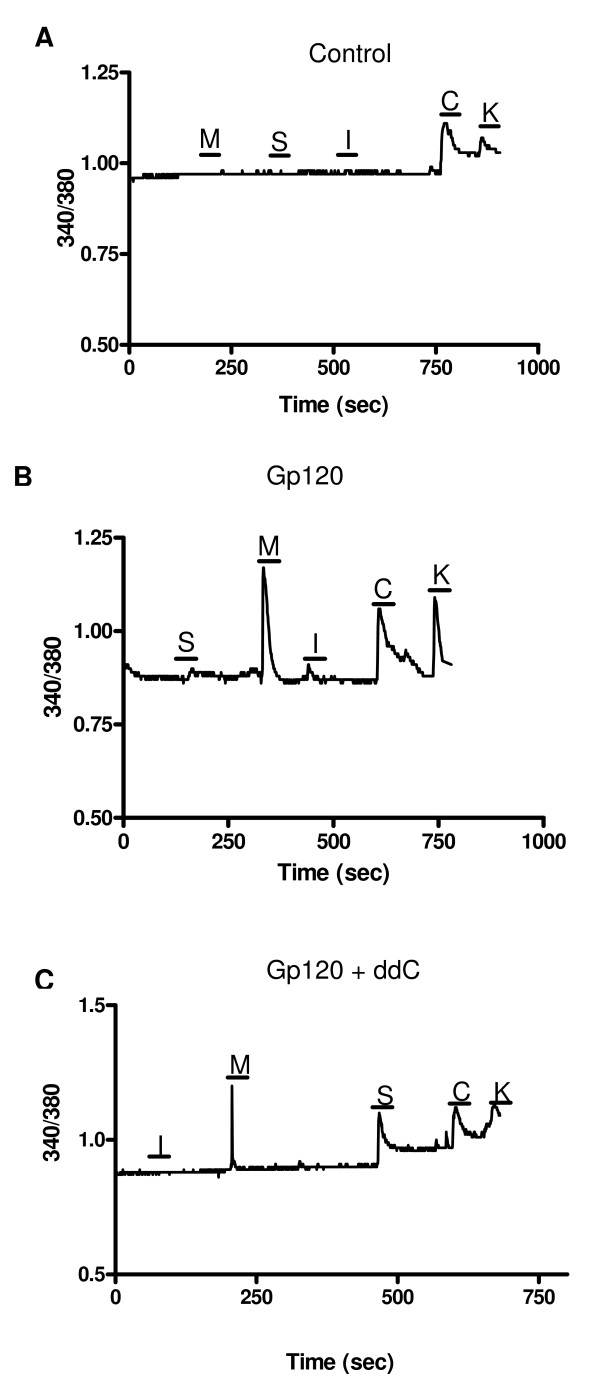
**Chemokines increased [Ca2+]i levels in acutely isolated rat DRG cells following the injury schemes**. The figure shows examples of responses of cells acutely isolated from rat DRGs ipsilateral to the gp120/hCD4 and following gp120/hCD4 and ddC nerve injury at POD 21. Under normal conditions, cells rarely responded to any chemokine, but did respond to other stimuli such as high K or capsaicin (**A**). However, there was an increased responsiveness of the cells, the majority of which could be characterized as neurons, by POD21 in both the gp120/hCD4 only (**B**) and the combination of gp120/hCD4 with ddC injuries (**C**). For all experiments, MCP1 (**M**), IP-10 (**I**) and SDF1 (**S**) were applied at a concentration of 100 nM. Capsaicin (**C**) and high K (**K**) were applied at concentrations of 100 nM, 50 mM, respectively.

It was evident that a greater number of cells responded to MCP1 application at POD 21 in the DRG ipsilateral to the perineural gp120/hCD4 injury when compared to vehicle treated control DRG (18.7% versus 3.2%, respectively; Fig 7A, B, and Table 1). The majority of these cells were characterized as neurons based on their positive responses to capsaicin and/or high K+ (Table 2). The number of cells responding to SDF1 application was 4.3%, and was not significantly different from sham animals. The number of cells responding to either SDF1 or IP-10 application did not change. IP-10, the ligand for the CXCR3 chemokine receptor, was not upregulated in this animal model (data not shown). Thus, the fura-2 imaging generally confirmed the observed upregulated CCR2 expression in sensory neurons following perineural gp120/hCD4 treatment.

**Table 1:** Percentage of cells responding to chemokine application in dissociated DRG ipsilateral to nerve injury at POD 21

**Chemokine**	**Sham**	**gp120/hCD4**	**gp120/hCD4 +ddC**
**MCP1**	3.2%	18.7%*	17.5% *

**SDF1**	4.8%	4.3%	40%**

**IP10**	6.5%	5.9%	7.5%

*p < 0.05 and ** p < 0.01 when compared to sham

Minimum of 50 cells/treatment group

Calcium imaging was used to monitor the change in [Ca^2+^]i after chemokines were applied to acutely dissociated DRG from sham or injured rats. The number of cells responding to MCP1 application increased significantly by 21 days after gp120/hCD4 and following the combination of gp120/hCD4 and ddC treatment. The number of cells responding to SDF1 application increased only after the combination of gp120/hCD4 and ddC treatments.

**Table 2:** Identity of cells responding to chemokine application at POD 21 after gp120/hCD4 treatment

**Capsaicin/High K+/ATP **positive (TRPV1 expressing nociceptor)	59.5%
**High K+/ATP **positive only (Non TRPV1-expressing neuron)	27.0%

**ATP **positive only (Non-neuronal cell)	13.5%

The chemokine sensitivity profile also changed after the rats were treated with the combination of gp120/hCD4 and ddC as the number of cells responding to SDF1 application at POD 21 increased from 4.8% in vehicle treated animals to 40% in cells derived from injured animals (Fig 7C, Table 1). The number of cells that responded to MCP1 in tissue derived from gp120/hCD4 and ddC treated animals did not differ from gp120/hCD4 treatment condition (17.5% and18.7%, respectively). Furthermore, most of the cells responding to MCP1 and SDF1 application were neurons, based on their response profile to capsaicin, high K+ and ATP (Table 3). Hence, the observed upregulation of responses to SDF1 and MCP1 application agreed with the anatomical observations on receptor expression described above.

**Table 3:** Identity of cells responding to chemokine application at POD 21 after the combination of gp120/hCD4 and ddC

**Capsaicin/High K+/ATP **positive (TRPV1 expressing nociceptor)	75.8%
**High K+/ATP **positive only (Non TRPV1-expressing neuron)	15.2%

**ATP **positive only (Non-neuronal cell)	9.0%

### Chemokine receptor antagonists transiently reverse mechanical hypernociceptive behavior after injury

**Figure 8:**
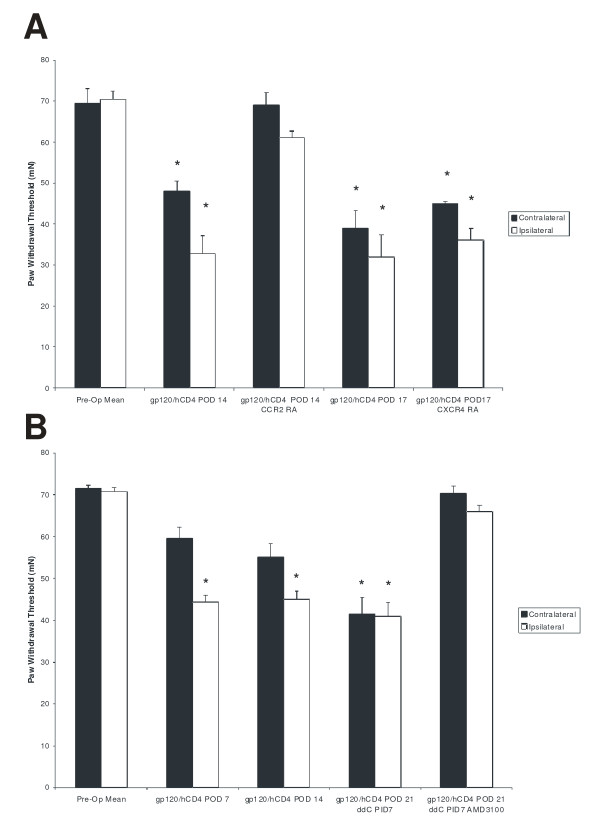
**Effect of CCR2 receptor antagonist (RA) and CXCR4 RA on gp120/hCD4 alone and following gp120/hCD4 and ddC-induced decrease in paw withdrawal thresholds (PWT) in the rat**. Mechanical hypernociception for the two models was attenuated with antagonists to chemokine receptors. **A) **CCR2 receptor antagonist administration attenuated existing nociceptive behavior due to gp120/hCD4 administration. In the same animals, intraperitoneal administration of the CXCR4 receptor antagonist, AMD3100, did not affect gp120/hCD4 induced mechanical hypernociception. **B) **Animals subjected to a combination of gp120/hCD4 and ddC exhibited significant increases in PWT following the administration of AMD3100. Data represent means ± SE; *p < 0.01, n = 10 per treatment condition. POD: post-operative day after gp120/hCD4 treatment; PID: post-injection day after ddC treatment.

The CCR2 receptor antagonist (CCR2 RA-**[R]**) and the CXCR4 receptor antagonist, AMD3100, have previously been shown to reverse mechanical hypernociception in two independent injury models; a focal demyelinating lesion (CCR2 RA-**[R]**) and, ddC treatment (AMD3100) [22,24]. To assess the ability of these receptor antagonists to reverse injury induced changes in behavioral responses we administered a single injection of CCR2 RA-**[R] **(i.p., 10 mg/kg) 14 days afterperineural gp120/hCD4 treatment. Treatment with CCR2 RA-**[R] **transiently reversed mechanical hypernociception (Fig. 8A). In the same group of animals, administration of the CXCR4 antagonist, AMD3100 (i.p., 5 mg/kg) at 17 days after perineural gp120/hCD4 treatment did not alter the presence of mechanical hypernociceptive behavior (Fig. 8A). In contrast, when administered to gp120/hCD4 and ddC treated animals at POD 21, AMD3100 (i.p., 5 mg/kg) transiently reversed mechanical hypernociceptive behavior, while administration of CCR2-RA-**[R] **(i.p., 10 mg/kg) produced no change in mechanical hypernociceptive behavior (Fig. 8B). The effect of the AMD3100 in gp120/hCD4 and ddC treated animals was similar to effect on animals treated only with ddC [22].

## Discussion

Previous studies have demonstrated the important role of chemokine signaling in neuropathic pain models [7,22,24,34]. It was previously shown that NRTI treatment alone resulted in both increased mechanical hypersensitivity and upregulation of CXCR4/SDF1 signaling in the DRG [22]. While this previous study was informative in beginning to understand the mechanisms underlying this type of neuropathic pain, we wished to observe the degree to which the HIV1 viral coat protein, gp120 interacts with ddC and if this changes the drug associated pattern of chemokine signaling and chronic nociceptive behavior. To accomplish this, NRTI treatment was combined with perineural exposure to gp120/hCD4. Importantly, the T-tropic HIV1 viral coat protein, gp120 IIIB, was primed with hCD4, which facilitates high affinity binding to CXCR4 chemokine receptors [17]. T-tropic versions of HIV1 generally emerge later in the course of the disease and it is possible that M-tropic versions would produce a different set of phenomena. However, there is no consensus at this point in time as to the correlation between viral tropism and the appearance of clinical neuropathic pain. Indeed, it is quite possible that both types of virus contribute to this phenomenon [35]. The model we have used was previously shown to result in mechanical hypernociception and reduced intraepidermal nerve fiber density, which is similar to what is seen in the clinical setting [10].

It was hypothesized that while administration of either an NRTI or gp120/hCD4 might result in upregulation of chemokine signaling, the combination of gp120/hCD4 and an NRTI might result in a synergistic effect. In rats following gp120/hCD4 treatment alone, mechanical hypernociception developed as early as POD 3 and lasted until at least POD 28. Together with behavioral changes, pronounced increases in MCP1/CCR2, but not SDF1/CXCR4 chemokine signaling events were observed. The role of enhanced MCP1/CCR2 signaling in gp120/hCD4 injury induced mechanical hypernociception was confirmed by the reversal of nociceptive behavior on treatment with a CCR2 RA. The combination of gp120/hCD4 and ddC produced mechanical hypernociception similar to gp120/hCD4 alone, along with enhanced MCP1/CCR2 and SDF1/CXCR4 signaling. However, following this treatment combination only AMD3100 a CXCR4 antagonist was effective in reversing mechanical hypernociception. Taken together, these results suggest a role for both SDF1/CXCR4 and MCP1/CCR2 signaling in HIV1/ddC associated mechanical hypernociceptive behavior (Table 4).

**Table 4:** Summary figure of gp120/hCD4 and NRTI treatments

	gp120/hCD4	ddC	gp120/hCD4+ddC
CXCR4	No change	↑	

SDF1	No change	↑	↑

CCR2	↑	No change	↑

MCP1	↑	No change	↑

Behavior	Mechanical	Mechanical, thermal	Mechanical, thermal

Several studies on the mechanisms underlying chronic pain have focused on the CCR2 and CXCR4 receptors, and their respective ligands, MCP1 and SDF1. This is because along with a previous study, where the importance of CXCR4/SDF1 signaling in a NRTI-associated neuropathic pain model was demonstrated, other studies had shown that peripheral nerve injury resulted in the upregulation of CCR2 and its ligand MCP1 in DRG neurons [7,22,24,36-38]. Indeed, administration of a CCR2 RA blocked mechanical hypernociception in a focal demyelination model in which both MCP1 and CCR2 expression were upregulated [24,38]. Furthermore, a study by Abbadie and colleagues [34] demonstrated that mice lacking the CCR2 receptor lack neuropathic pain impairment after injury.

We observed that after gp120/hCD4 treatment, MCP1/CCR2 signaling events were upregulated in neurons of the affected DRG. It has been shown that MCP1 has an excitatory effect on DRG sensory neurons after injury [7]. Work from our laboratory has also shown MCP1 can be packaged into secretory vesicles and released upon depolarization [39]. Thus, MCP1 could be released by DRG sensory neurons and affect neighboring, uninjured CCR2 expressing neurons increasing their excitability [7]. It is believed that chemokines can produce their excitatory effect by activating the phospholipase C-induced degradation of PIP2, which would then lead to the transactivation of TRPV1 channels [39,40]. In addition, activation of chemokine receptors clearly has excitatory actions mediated through effects on Na and K currents [6]. Thus, MCP1 may potentially excite TRPV1 expressing as well as other nociceptive neurons. Alternatively, MCP1 immunopositive fibers in the spinal cord dorsal horn may release the chemokine and activate neurons and/or non-neuronal cells [37,41,42].

The increased mechanical hypersensitivity seen here agrees with previous studies, where it was shown that peripheral nerve exposure to gp120 (albeit without hCD4) resulted in mechanical hypersensitivity for up to 50 postoperative days, with a lack of thermal or cold hyperalgesia [9,15]. While the lack of thermal behavior is unusual for a rodent neuropathic pain models, several models do indeed lack thermal hyperalgesia, including a fibromyalgia model [43,44] and focal demyelination of the sciatic nerve [24]. Perhaps more importantly, there are a large percentage of patients with HIV-1 associated neuropathic pain who do not report thermal hyperalgesia [45].

There have been several mechanisms suggested for explaining the deleterious effects of HIV-1 on sensory neuron function. gp120, independent of the conformational change by hCD4, produces neuritic and axonal degeneration of DRG neurons in culture via a chemokine receptor dependent mechanism [10,46]. It was also observed that the use of gp120 (without the addition of hCD4) produced the release of proinflammatory cytokines such as TNFα, IL-6 and IL1-β [14,15]. Whether the addition of hCD4 induced conformational changes to gp120 augments these inflammatory responses in the rodent is unknown. However, the combination of the gp120 induced cascades of pro-inflammatory cytokine production and release likely lead to both the development of neuropathic hypernociceptive behavior in the rodent and chronic expression of nociceptive chemokines [47].

The combination of a T-tropic form of gp120 with hCD4 did not alter the expression or functional signaling of SDF1/CXCR4 in the DRG, however, increased expression of both MCP1 and CCR2 was observed, in addition to increased functional signaling via CCR2. The changes in MCP1 resemble those observed by Wallace and colleagues (2007) using gp120 alone as well as those observed in other injury models [7,24]. The increased functional signaling of MCP1/CCR2 may be due to an indirect mechanism by which CXCR4 receptors located on Schwann cells in the peripheral nerve are activated by the gp120/hCD4. These cells may release RANTES within the perineural environment and release of TNFα in the DRG [10]. As mentioned previously, TNFα could then initiate an "inflammatory" cascade and increased neuronal expression of MCP1 and CCR2. Hence, the importance of MCP1/CCR2 signaling following administration of a T-tropic gp120/hCD4 may involve a hierarchy of effects wherein initial interactions with CXCR4 lead to downstream changes in MCP1/CCR2 functional signaling. It should be noted that direct gp120/hCD4 signaling via CXCR4 was not the causative effect, as AMD3100 did not alter hypernociceptive behavior in this model.

Also, consistent with this model, administration of a CCR2 RA was effective in transiently eliminating the mechanical hypernociceptive behavior observed after gp120/hCD4 treatment. This could be due to the fact that upregulation of chemokine receptors can be expressed in different populations of sensory neurons following nerve injury [48,49]. In our experiments up to 57% of the cells that upregulated CCR2 were positive for IB_4_, a marker of non-peptidergic C-fiber nociceptors. Nearly the same numbers of CCR2 immunopositive neurons were colocalized with a nonoverlapping peptidergic population of nociceptors that exhibited the cation channel, TRPV1. This is an important observation as many groups believed that the reversal of injury induced mechanical hypernociceptive behavior observed with either the use of mice lacking CCR2 or CCR2 RA was mechanistically due to CCR2 bearing non-neuronal cells in the CNS as microglial cells [34,42,50,51]. A more recent publication provides direct evidence that CCR2 expression is limited to the DRG following peripheral nerve injury [38].

An important goal of this series of experiments was to model the neuropathic pain syndromes reported by HIV-1 positive individuals being treated with NRTIs. To do this, the perineural gp120/hCD4 injury was combined with a known neurotoxic NRTI, ddC. We have previously shown that ddC treatment alone resulted in the upregulation of CXCR4 and SDF1 mostly in glia, but also in some neurons of the DRG. Despite a report to the contrary [15], we did not observe increased expression of either MCP1 or the CCR2 following ddC treatment. This discrepancy could be due to a difference in ddC dosage or other unidentified factors. However, when both treatments were combined, rather than observing the increased expression of either MCP1/CCR2 or SDF1/CXCR4, both chemokine signaling systems were upregulated and this was observed to occur mostly in neurons. Moreover, the degree to which CXCR4 upregulated was much greater than that observed following ddC treatment alone. This synergistic effect of the two independent treatments is interesting, and may mechanistically facilitate chronic maintenance of mechanical hypernociception in the rodent and clinical neuropathic pain reported in HIV-1 associated peripheral neuropathy.

The combination of perineural gp120/hCD4 treatment with ddC produced increased neuronal expression of both CXCR4 and CCR2 receptors together with their respective ligands, SDF1 and MCP1 in rather distinct subpopulations of sensory neurons. Almost equal numbers of chemokine receptor immunopositive neurons colocalized with either IB_4 _or TRPV1; both of which are mutually exclusive markers of nociceptors [48,49]. As such, it is quite possible that the population of CCR2 or CXCR4 positive neurons is directly linked to the production of chronic neuronal hyperexcitability.

Perhaps a more important observation is that following the combination of the perineural gp120/hCD4 treatment with the NRTI treatment, mechanical hypernociceptive behavior in the rodent became a CXCR4 receptor dependent phenomenon. In the combined treatment model, the level of SDF1/CXCR4 signaling increased from 4% (under sham or gp120/hCD4 treatment condition) to 40%. Concurrent with the change in SDF1/CXCR4 functional signaling was the ability of the CXCR4 antagonist AMD3100 to effectively produce a transient reversal of mechanical hypernociceptive behavior. This CXCR4 dependent effect occurred despite the presence of ongoing injury induced MCP1/CCR2 signaling.

The behavioral effect of AMD3100 under the conditions of both injury induced CCR2 and CXCR4 functional signaling may be due to the possibility that chemokine receptors under these conditions may form homo- and heterodimers, which may ultimately alter their signaling properties. For example, it was recently demonstrated that CCR2 and CXCR4 could form heterodimers resulting in receptors with distinct properties [52]. Furthermore, a specific antagonist to one of the receptors was observed to inhibit binding of a chemokine ligand to the partner receptor *in vitro *and *in vivo *[53]. These results have important implications in the present pain model, where an antagonist to the CXCR4 receptor was effective in completely attenuating the pain behavior despite the fact that functional CCR2 receptors were simultaneously upregulated.

Another difference between gp120/hCD4 alone or in combination with the ddC was the observed thermal hypernociceptive behavior in the combination injury. It was previously shown that administration of ddC alone resulted in an increased hypersensitivity to a thermal stimulus [54], yet gp120 (without the addition of hCD4) treatment alone did not result in thermal hyperalgesic behavior [15]. Therefore, our results are consistent with these previous studies. One possible mechanism that has been suggested for the additive effect of the ddC is that this compound quite often results in mitochondrial dysfunction. Studies have also shown that a number of mitochondrial abnormalities have been linked to neuropathic pain symptoms [54,55].

In summary, using a combined model of NRTI and HIV-1 associated peripheral neuropathy, we have demonstrated that two different chemokine signaling systems may interact and result in the maintenance of this neuropathic pain syndrome. These results, taken with previous studies, suggest an important role for the SDF1/CXCR4 and MCP1/CCR2 chemokine signaling systems in HIV-1 associated painful neuropathies and are perhaps potential therapeutic targets.

## Competing interests

The authors declare that they have no competing interests.

## Authors' contributions

SKB, RJM and FAW designed research. SKB, MSR and DB performed research. SKB FAW analyzed data. SKB, RJM and FAW wrote the paper. All authors read and approved the final manuscript.

## Supplementary Material

Additional file 1 **CXCR4 and CCR2 chemokine receptor immunoreactivity in naïve animals**. CCR2 receptor protein (**A**) and mRNA (**C**) expression levels are absent from lumbar DRG taken from naïve rats. Unlike the CCR2 receptor, the CXCR4 chemokine receptor is constitutively expressed in naïve DRG. CXCR4 protein (**B**) and mRNA (**D**) expression levels are present in mainly non-neuronal cells in naive lumbar DRG. Click here for file
