# Supplementary figures and images for: Increased chemokine signaling in a model of HIV1-associated peripheral neuropathy
Source: Mol Pain. 2009 Aug 12;5:48. doi: 10.1186/1744-8069-5-48 (PMC2734548; doi:10.1186/1744-8069-5-48)

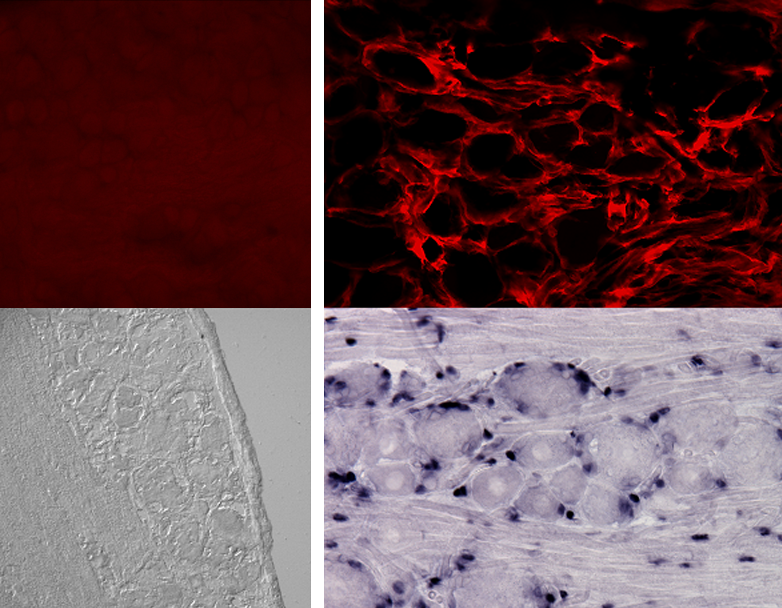


**C**

**D**

**A**

**B**

**C**

**D**

Supplement: Additional file 1 — CXCR4 and CCR2 chemokine receptor immunoreactivity in naïve animals. CCR2 receptor protein (A) and mRNA (C) expression levels are absent from lumbar DRG taken from naïve rats. Unlike the CCR2 receptor, the CXCR4 chemokine receptor is constitutively expressed in naïve DRG. CXCR4 protein (B) and mRNA (D) expression levels are present in mainly non-neuronal cells in naive lumbar DRG. [file 1744-8069-5-48-S1.doc]
